# Supplementary material for: Vascular and blood-brain barrier-related changes underlie stress responses and resilience in female mice and depression in human tissue
Source: Nat Commun. 2022 Jan 10;13:164. doi: 10.1038/s41467-021-27604-x (PMC8748803; doi:10.1038/s41467-021-27604-x)
Supplement: Supplementary file 1 — Supplementary Information [file 41467_2021_27604_MOESM1_ESM.pdf]

Supplementary Information

Vascular and blood-brain barrier-related changes underlie stress responses and resilience in female mice and depression in human tissue

Laurence Dion-Albert[1], Alice Cadoret[1], Ellen Doney[1], Fernanda Neutzling Kaufmann[1], Katarzyna A. Dudek[1], Beatrice Daigle[1], Lyonna F. Parise[2], Flurin Cathomas[2], Nalia Samba[3], Natalie Hudson[4], Manon Lebel[1], Signature Consortium[5], Matthew Campbell[4], Gustavo Turecki[6], Naguib Mechawar[6], Caroline Menard[1]*

[1]Department of Psychiatry and Neuroscience, Université Laval and CERVO Brain Research, Quebec City, QC (Canada)

[2]Fishberg Department of Neuroscience and the Friedman Brain Institute, Icahn School of Medicine at Mount Sinai, New York, NY, USA

[3]Sorbonne Université, Paris (France)

[4]Smurfit Institute of Genetics, Trinity College Dublin, Lincoln Place Gate, Dublin 2 (Ireland)

[5]Institut universitaire en santé mentale de Montréal, Centre intégré universitaire de santé et service sociaux Est, Montreal, QC (Canada)

[6]Department of Psychiatry, McGill University and Douglas Mental Health University Institute, Montreal, QC (Canada)

Corresponding author: ***Caroline Menard**, PhD, CERVO Brain Research Center, Department of Psychiatry and Neuroscience, Faculty of Medicine, Université Laval, 2601 de la Canardiere, Quebec City, QC, Canada, G2J 2G3, caroline.menard@fmed.ulaval.ca

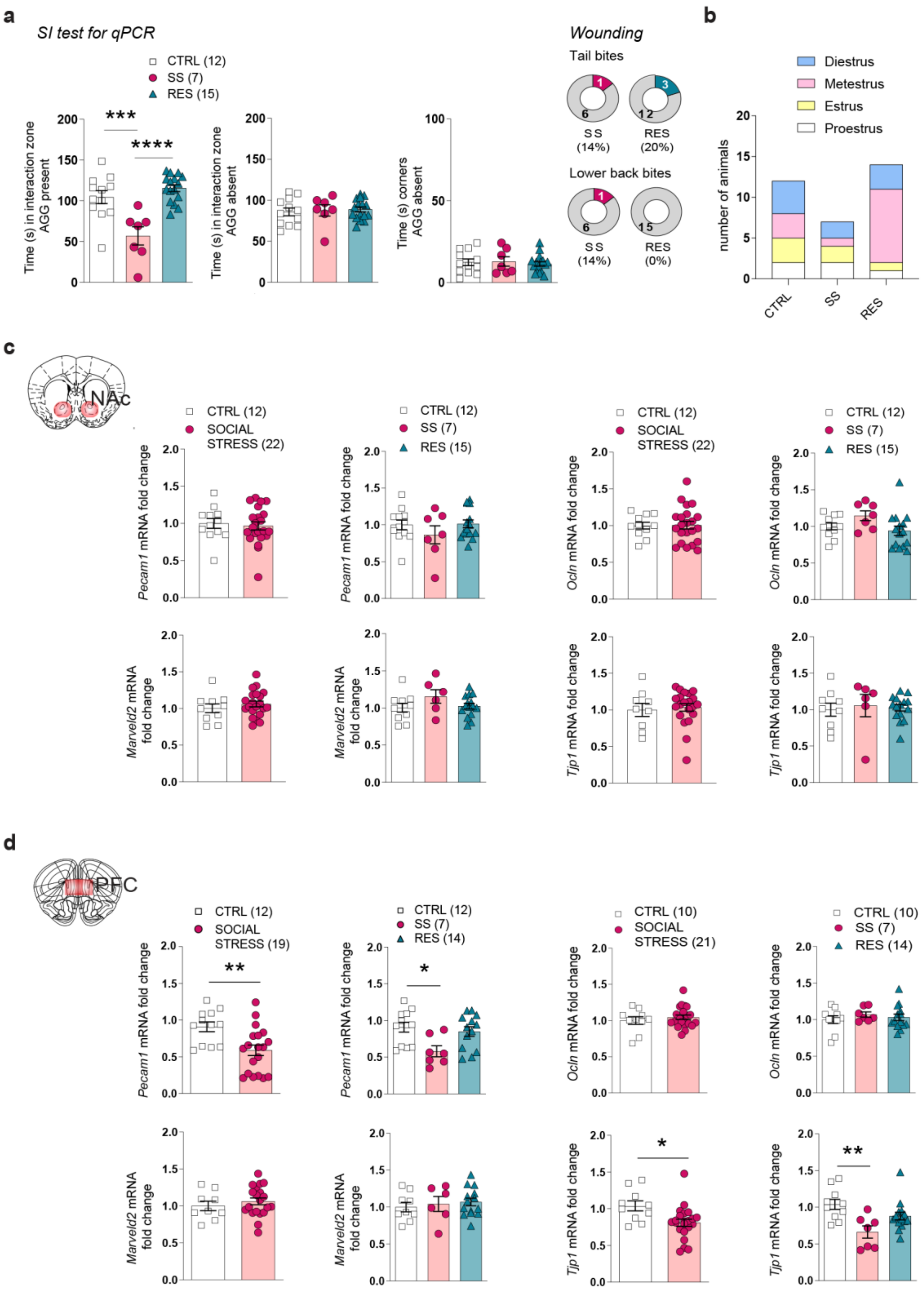

Supplementary Figure 1. Behavioural phenotype of quantitative PCR (qPCR) experiments and *Cldn5* expression in the nucleus accumbens (NAc) and prefrontal cortex (PFC) of female

mice after chronic social defeat stress (CSDS). a, Stress susceptible (SS) mice spent less time in the interaction zone when the social target (aggressor, AGG) was present compared to unstressed control (CTRL) and resilient (RES) animals (****$p<0.0001$). No significance was observed for time in the interaction zone or in the corners when the social target is absent. Wounding was comparable between stressed groups with 20% or less animals with tail or lower back bites. **b**, No significant difference was observed between phenotype and phase of the estrus cycle at the time of tissue collection. **c,** Following 10-d CSDS, no difference was observed in *Pecam1, Ocln, Marveld2 or Tjp1* mRNA expression in the NAc of female mice, (**d**) while decreased expression of *Pecam1* (**$p=0.0048$ CTRL vs SOCIAL STRESS; * $p=0.0149$ CTRL vs SS vs RES) and *Tjp1* (**$p=0.0083$ CTRL vs SOCIAL STRESS; * $p=0.0106$ CTRL vs SS vs RES) mRNA levels were observed in the PFC of stressed mice. Data represent mean ± s.e.m; number of animals or subjects (*n*) is indicated on graphs. 2-group comparisons were evaluated with unpaired t-tests, two-way ANOVA followed by Bonferroni's multiple comparison test for estrus cycles (phase x phenotype) and one-way ANOVA followed by Bonferroni's multiple comparison test for other graphs. *$p<0.05$; **$p<0.01$; ***$p<0.001$, ****$p<0.0001$. Source data are provided as a Source Data file.

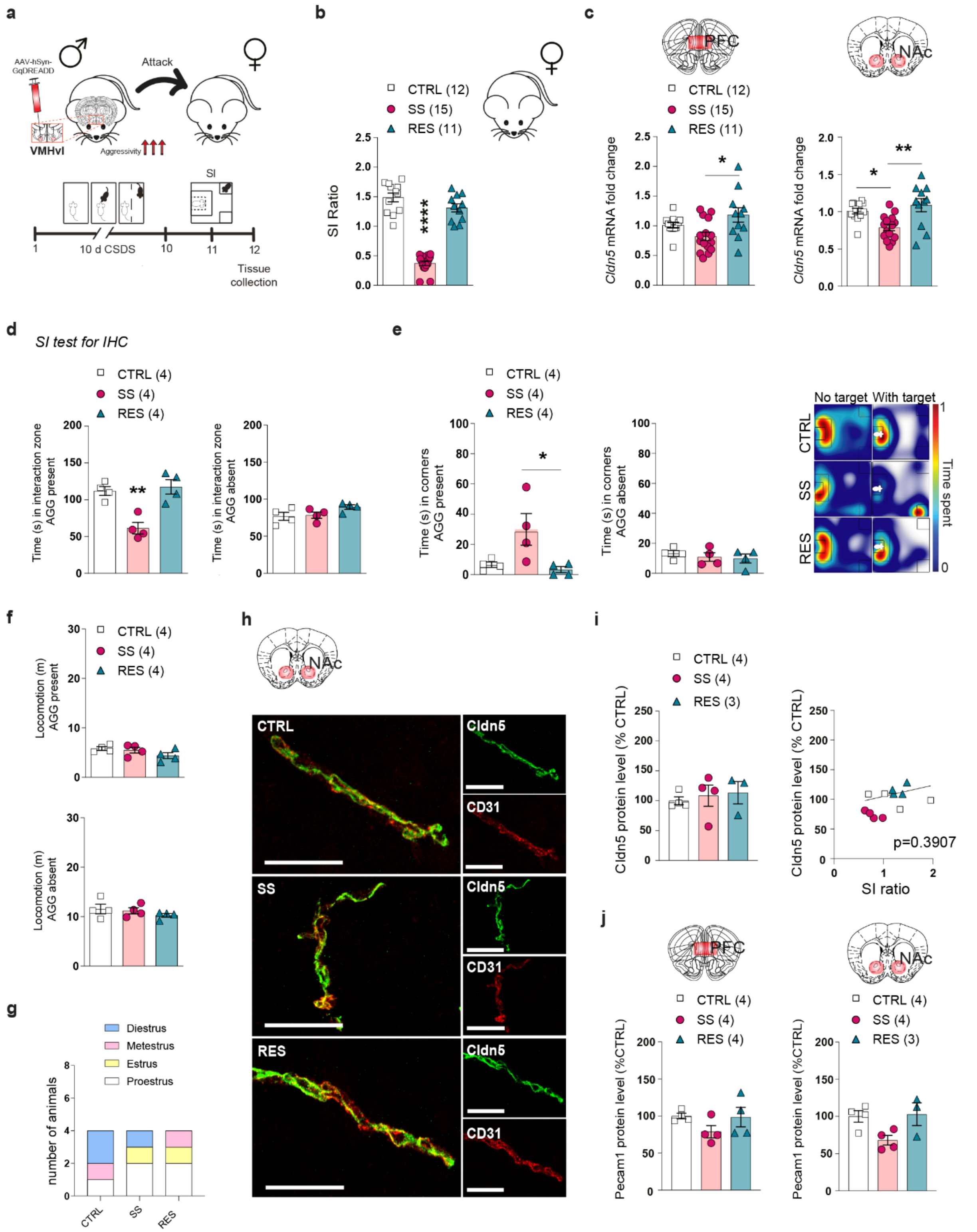

Supplementary Figure 2. *Cldn5* expression is altered in the nucleus accumbens (NAc) and prefrontal cortex (PFC) of female mice after chronic social defeat stress (CSDS) using

hyperaggressive males however no significant change was observed at protein level in the NAc. a, Schematic representation of the CSDS female paradigm developed by Takahashi et al. (2017). **b,** Like in the conventional male paradigm, 10-d physical exposure to hyperaggressive CD1 males (aggressor, AGG) leads to two female subpopulations – stress-susceptible (SS) and resilient (RES) - as defined by the social interaction (SI) test (****$p<0.0001$). **c,** Stress susceptibility was associated with a loss of *Cldn5* expression in the PFC (*$p=0.0122$) and NAc (**$p=0.0013$) of female mice (normalized on *Pecam1)* in this CSDS model. **d,** Behavioural phenotyping of the female cohort used for immunohistochemistry with SS mice spending less time in the interaction zone (**$p=0.0013$) and (**e**) increased time in the corners when the social target was present compared to unstressed control (CTRL) and RES animals (*$p=0.0292$). No significant difference was observed when the social target was absent. **f,** No difference was observed in total travelled distance (m) either when the social target was present or absent. **g,** No significant difference was observed between phenotype and phase of the estrus cycle at the time of tissue collection. **h,i,** Cldn5 protein levels in the NAc were not significantly different between CTRL, SS and RES animals, and do not correlate with social avoidance ($p=0.3907$). Scale bars, 20µm. **j,** No difference was observed in protein levels of endothelial marker Pecam1 in the PFC or NAc of animals. Data represent mean ± s.e.m; number of animals or subjects (*n*) is indicated on graphs. 2-group comparisons were evaluated with unpaired t-tests, two-way ANOVA followed by Bonferroni's multiple comparison test for estrus cycles (phase x phenotype) and one-way ANOVA followed by Bonferroni's multiple comparison test for other graphs. *$p<0.05$; **$p<0.01$. Source data are provided as a Source Data file.

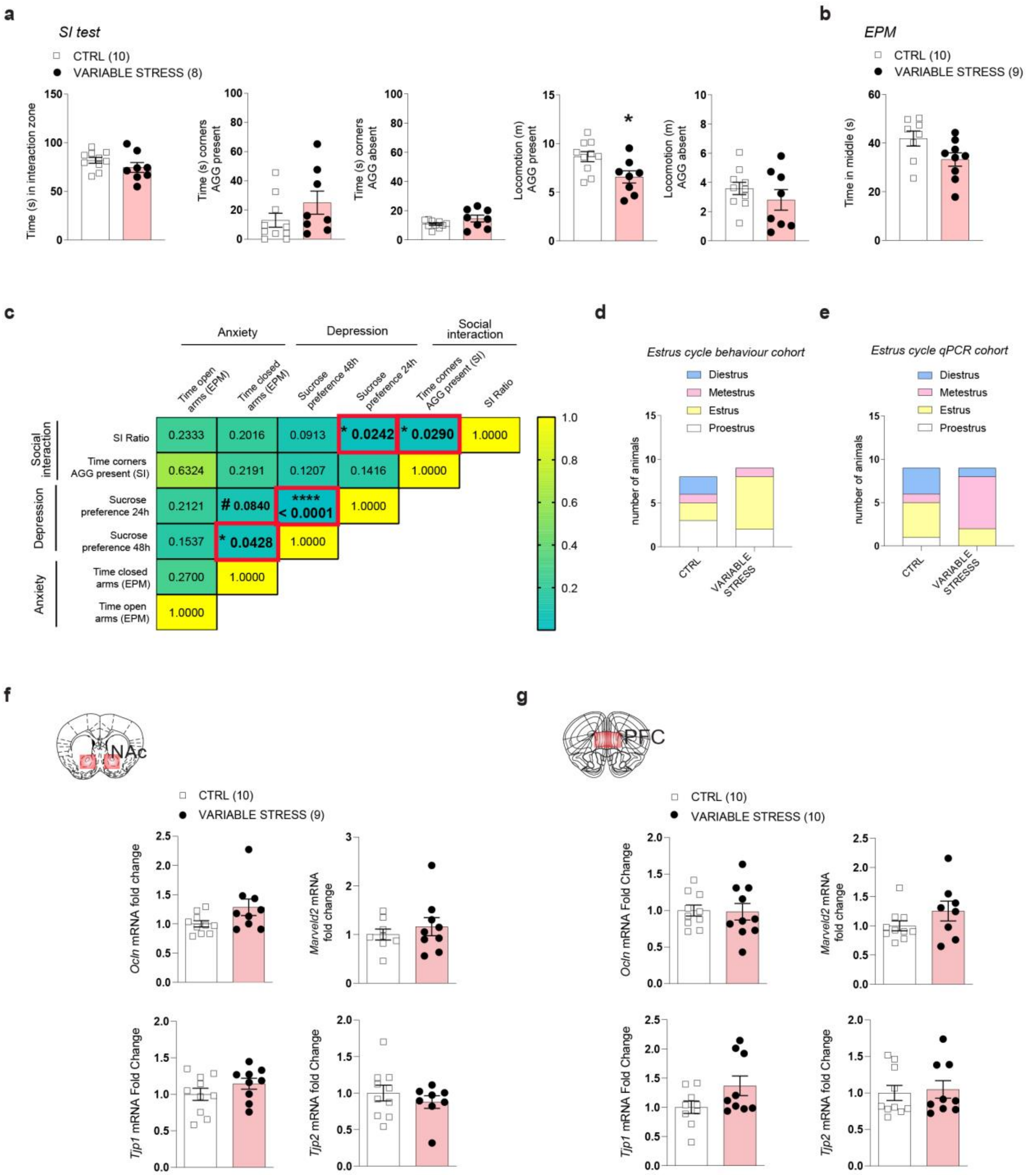

Supplementary Figure 3. Behavioural phenotypes and quantitative PCR (qPCR) experiments in the nucleus accumbens (NAc) and prefrontal cortex (PFC) of female mice after 6d subchronic variable stress (SCVS). a, following 6-d SCVS, variable stress and control (CTRL) females spent similar times in the interaction zone and corners when the social target

(aggressor, AGG) was present or absent. SCVS females travelled less distance when the aggressor was present (*p=0.0202) vs CTRL. **b,** No significant difference was observed for time in the middle of the EPM between stressed and CTRL animals. **c,** Intraindividual correlations between various behavioural data points shows association between social interaction, anxiety- and depressive-like behaviours. P values in the boxes refer to the strength of the correlation between behaviours. **d,e,** No significant difference was observed between phenotype and phase of the estrus cycle between CTRL and variable stress animals in the behaviour (**d**) and qPCR (**e**) cohorts. **f,g,** Following 6-d SCVS, no difference was observed in *Ocln, Marveld2, Tjp1, or Tjp2* mRNA levels in the NAc (**f**) or PFC (**g**) of stressed female mice. Data represent mean ± s.e.m; number of animals or subjects (*n*) is indicated on graphs. 2-group comparisons were evaluated with unpaired t-tests, two-way ANOVA followed by Bonferroni's multiple comparison test for estrus cycles (phase x phenotype) and one-way ANOVA followed by Bonferroni's multiple comparison test for other graphs. #$p<0.1$; *$p<0.05$; ****$p<0.0001$. Source data are provided as a Source Data file.

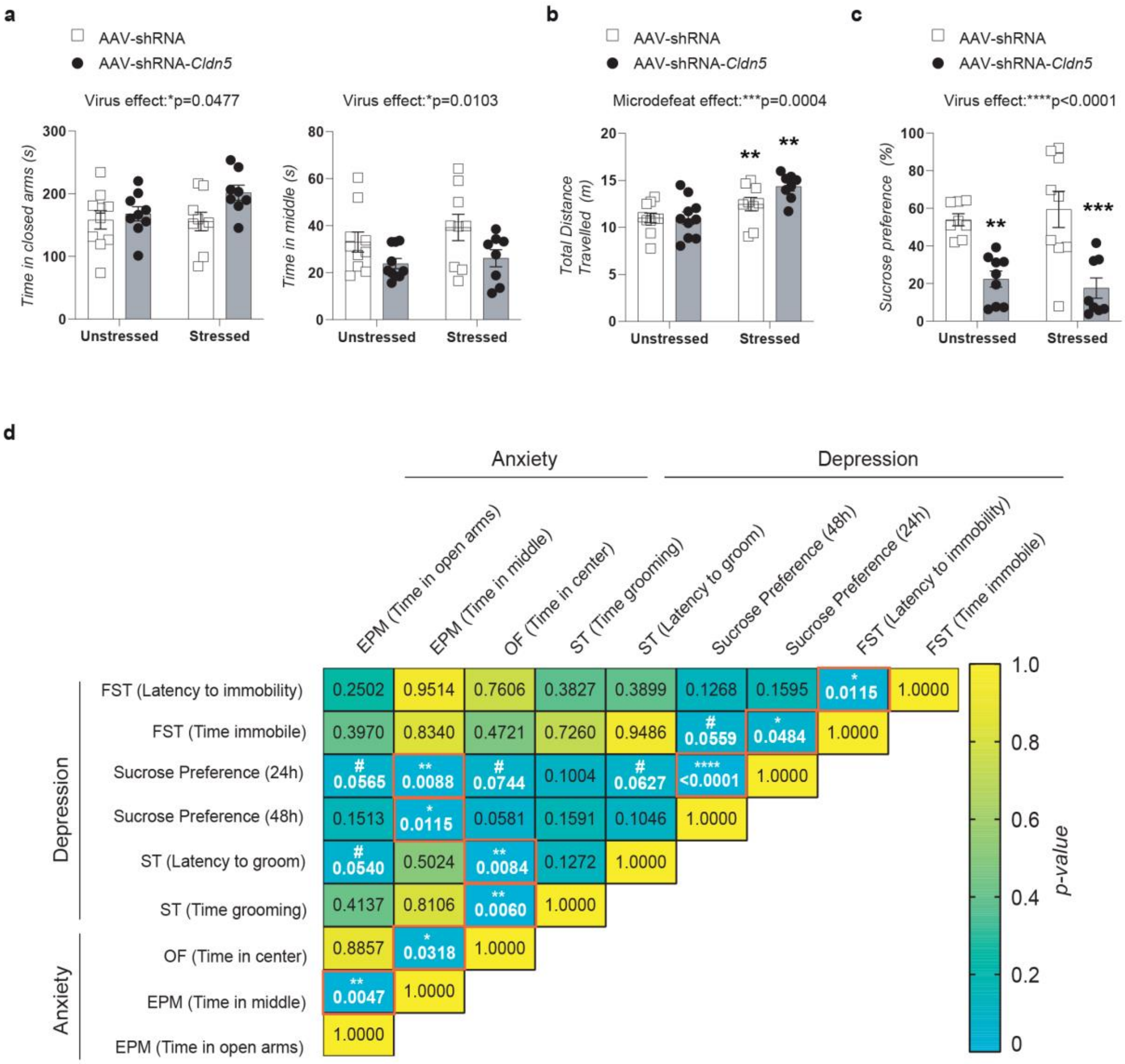

Supplementary Figure 4. Supplementary behaviour results for AAV-shRNA-*Cldn5* injected mice. a, A main virus effect was revealed in AAV-shRNA vs AAV-shRNA-*Cldn5* injected mice for time in closed arms (*p=0.0477) and middle area (*p =0.0103) in the elevated plus maze (EPM) test. **b,** Animals who underwent subthreshold microdefeat (stressed mice) travelled significantly higher distance than their unstressed counterparts (***p=0.0004). **c**, A highly significant main virus effect was found at 48h in the sucrose preference test (****p<0.0001; **p=0.0083 Unstressed AAV-shRNA vs AAV-shRNA-Cldn5; ***p=0.0003 Stressed AAV-shRNA vs AAV-shRNA-Cldn5). **d,** Intraindividual correlations between various behavioural data points shows

strong association between social interaction, anxiety- and depressive-like behaviour between AAV-shRNA and AAV-shRNA-*Cldn5* animals. P values in the boxes refer to the strength of the correlation between behaviours. Data represent mean ± s.e.m; number of animals or subjects (*n*). Correlations were evaluated with Pearson's correlation coefficient and two-way ANOVA followed by Bonferroni's multiple comparison test for other graphs. #$p<0.1$; *$p<0.05$; **$p<0.01$; ***$p<0.001$; ****$p<0.0001$. Source data are provided as a Source Data file.

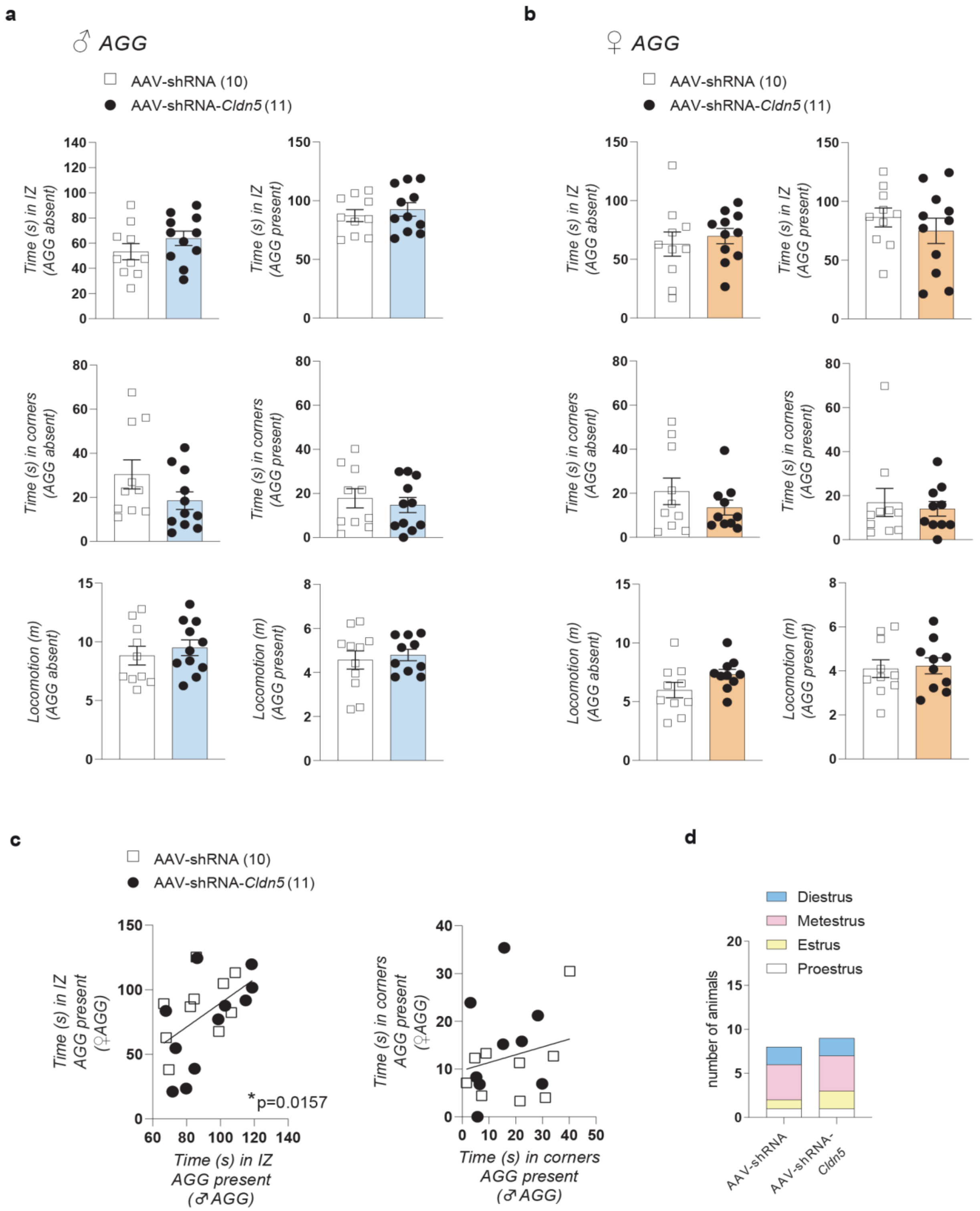

Supplementary Figure 5. Supplementary SI behaviour results for AAV-shRNA and AAV-shRNA-*Cldn5* injected mice. a,b, Time in the interaction zone (IZ), corners and total distance

travelled was not significantly different between AAV-shRNA and AAV-shRNA-*Cldn5*-injected mice, whether the male (**a**) or female (**b**) social target (aggressor, AGG) was present or absent. **c**, Time in the IZ when the female AGG was present significantly correlated to the time in the IZ when the male AGG was present (*p=0.0157), but no correlation was found for time in corners. **d,** A significant effect is observed between AAV-shRNA and AAV-shRNA-*Cldn5* injected animal (*p=0.0109). Data represent mean ± s.e.m; number of animals or subjects (*n*) is indicated on graphs. Correlations were evaluated with Pearson's correlation coefficient, 2-group comparisons were evaluated with unpaired t-tests, two-way ANOVA followed by Bonferroni's multiple comparison test for estrus cycles (phase x phenotype) or one-way ANOVA followed by Bonferroni's multiple comparison test for other graphs. *$p<0.05$. Source data are provided as a Source Data file.

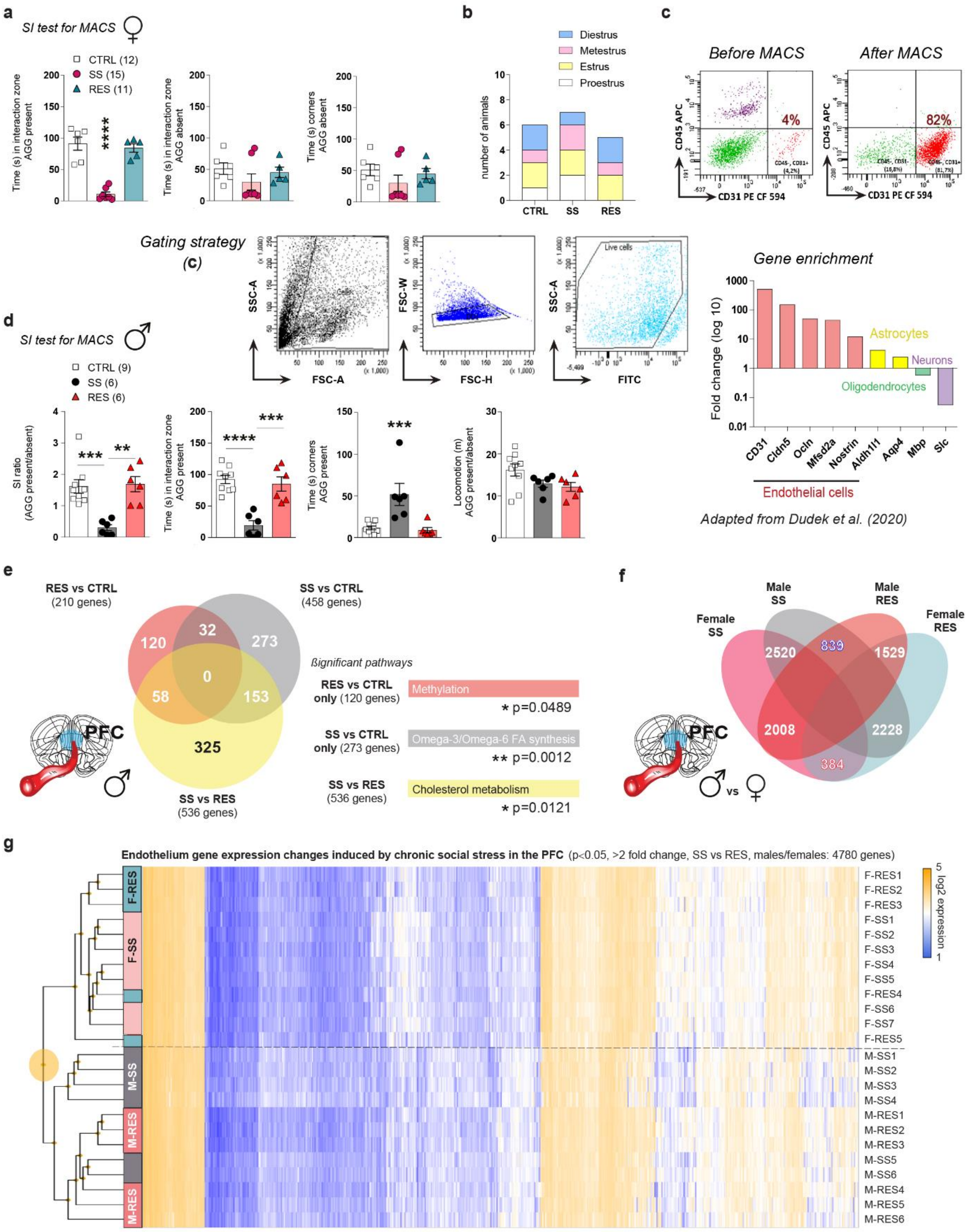
a
SI test for MACS ♀
CTRL (12)
SS (15)
RES (11)
Time (s) in interaction zone AGG present
Time (s) in interaction zone AGG absent
Time (s) corners AGG absent
****
b
Diestrus
Metestrus
Estrus
Proestrus
number of animals
CTRL
SS
RES
c
Before MACS
After MACS
CD45 APC
CD31 PE CF 594
4%
82%
Gating strategy (c)
SSC-A
FSC-A
FSC-W
FSC-H
FITC
Live cells
Gene enrichment
Fold change (log 10)
Astrocytes
Neurons
Oligodendrocytes
CD31
Cldn5
Ocln
Mfsd2a
Nostrin
Aldh1l1
Aqp4
Mbp
Slc
Endothelial cells
Adapted from Dudek et al. (2020)
d
SI test for MACS ♂
CTRL (9)
SS (6)
RES (6)
SI ratio (AGG present/absent)
Time (s) in interaction zone AGG present
Time (s) corners AGG present
Locomotion (m) AGG present/absent
***
**
****
e
RES vs CTRL (210 genes)
SS vs CTRL (458 genes)
SS vs RES (536 genes)
120
32
273
0
58
153
325
PFC
Significant pathways
RES vs CTRL only (120 genes)
Methylation
* p=0.0489
SS vs CTRL only (273 genes)
Omega-3/Omega-6 FA synthesis
** p=0.0012
SS vs RES (536 genes)
Cholesterol metabolism
* p=0.0121
f
Female SS
Male SS
Male RES
Female RES
2520
839
1529
2008
2228
384
♂ vs ♀
g
Endothelium gene expression changes induced by chronic social stress in the PFC (p<0.05, >2 fold change, SS vs RES, males/females: 4780 genes)
F-RES
F-SS
M-SS
M-RES
F-RES1
F-RES2
F-RES3
F-SS1
F-SS2
F-SS3
F-SS4
F-SS5
F-RES4
F-SS6
F-SS7
F-RES5
M-SS1
M-SS2
M-SS3
M-SS4
M-RES1
M-RES2
M-RES3
M-SS5
M-SS6
M-RES4
M-RES5
M-RES6
log2 expression

Supplementary Figure 6. Behavioural phenotyping of females subjected to CSDS for transcriptomic experiments, validation of magnetic-activated cell sorting (MACS) and comparison of stress- induced PFC endothelium transcriptomes for males vs females. a, Stress susceptible female (SS) mice spent less time in the interaction zone, when the social target (aggressor, AGG) was present compared to unstressed control (CTRL) and resilient (RES) animals during the social interaction (SI) test (*****p*<0.0001). No significance was observed for time in the interaction zone or in the corners when the social target is absent. **b**, No significant trend was observed between phenotype and phase of the estrus cycle at the time of tissue collection. **c**, Enrichment of endothelial cells following MACS was confirmed using flow cytometry and qPCRs (adapted from Dudek et al.[8]). As shown on the left, CD31+ cell population increases from ~4% in the heterogenous preparation to >80% after MACS. Gating strategy of flow cytometry is shown on the bottom left. Endothelial cell-specific gene expression (CD31*;* Claudin-5, *Cldn5;* Occludin, *Ocln;* Major facilitator superfamily domain-containing protein 2, *Mfsd2a; Nostrin*) is highly increased as well when compared to genes related to astrocytes (Aldehyde dehydrogenase 1 family member l1, *Aldh1l1;* Aquaporin 4, *Aqp4*), oligodendrocytes (Myelin basic protein, *Mbp*) or neurons (Solute carrier, *Slc*). **d**, Male SS mice are characterized by lower SI ratio (****p*=0.0003) and time spent in the interaction zone when the AGG is present (*****p*<0.0001) when compared to CTRL or RES animals following 10 days of chronic social defeat stress. Conversely, time spent in the corners is increased for this group (****p*=0.0005) with no significant difference noted for locomotion. **e**, Venn diagrams indicate poor overlap of gene expression changes in the male PFC when group comparisons were performed with the largest number of genes associated to SS vs RES animals. Most significant biological pathways for each group comparison are displayed on the right according to the group comparison color. **f**, Venn diagrams also indicate a poor overlap

of PFC gene expression changes between stressed males and females. **g**, Hierarchical clustering heatmap of SS and RES males and females revealed that sex is a more important factor for the PFC endothelium transcriptome after exposure to chronic social stress than the resulting behavioural phenotype (orange circle, significance was set at ±2-fold change and $p<0.05$). Data represent mean ± s.e.m; number of animals or subjects (*n*) is indicated on graphs. Two-way ANOVA followed by Bonferroni's multiple comparison test was used for estrus cycles (phase x phenotype), and one-way ANOVA followed by Bonferroni's multiple comparison tests for other graphs. *$p<0.05$; **$p<0.01$; ***$p<0.001$; ****$p<0.0001$. Source data are provided as a Source Data file.

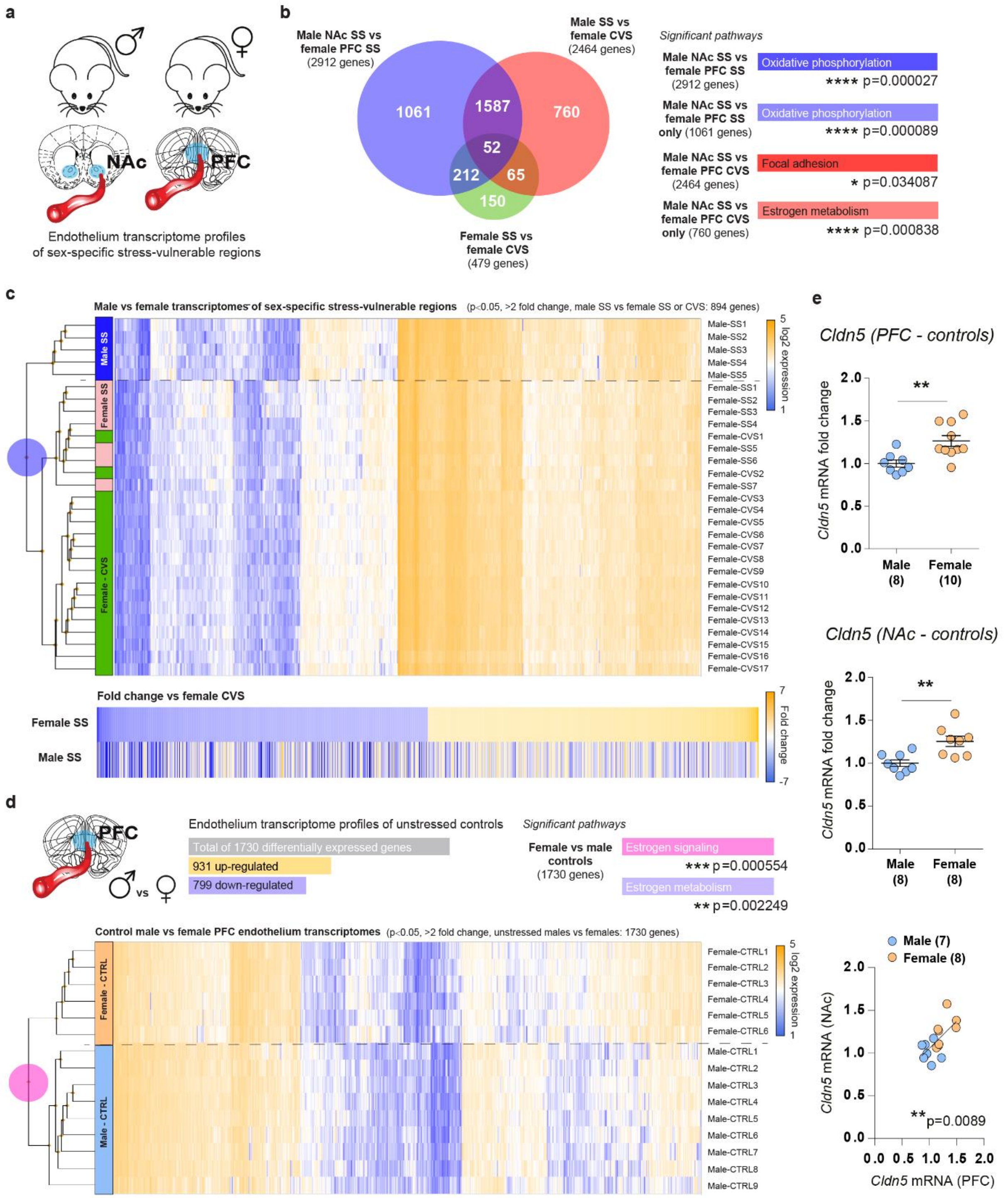

Supplementary Figure 7. Comparison of stress-induced transcriptomic changes in endothelial cells in male vs female's most vulnerable brain region and unstressed controls. **a,** Schematic outline of comparison of endothelial transcriptomic changes between male (nucleus

accumbens, NAc) and female (prefrontal cortex, PFC) brain regions characterized by stress-induced loss of blood-brain barrier integrity. **b,** Venn diagrams reveals poor overlap of gene expression changes when group comparisons were performed, with stress-susceptible (SS) male vs SS female showing the highest number of differentially regulated genes following 10 days of chronic social defeat stress (CSDS). Pathways related to these genes are displayed on the right. **c,** Hierarchical clustering indicates more genes regulated in the same direction between females exposed to different stress paradigms (CSDS vs CVS) then males vs females vulnerable to the same CSDS stressor (SS only). Significance was set at $p<0.05$ and ±2-fold change from the mean. **d**, Analysis of the endothelium transcriptome of the same brain region (PFC) in absence of stress (unstressed controls, CTRL) confirms that sex affects baseline endothelial gene expression (931 up-regulated and 799 down-regulated) with most genes being associated with estrogen signaling and metabolism. Significance was set at $p<0.05$ and ±2-fold change from the mean. **e,** Stress-naïve female mice exhibit higher *Cldn5* mRNA levels in the prefrontal cortex (PFC) (**p=0.0044) and nucleus accumbens (NAc) (**p=0.0034) than males, which are positively correlated with each other (**p=0.0089). Data represent mean ± s.e.m; number of animals or subjects (n) is indicated on graphs. Correlations were evaluated with Pearson's correlation coefficient and 2-group comparisons were evaluated with unpaired t-tests. *$p<0.05$; ** $p<0.01$; ***$p<0.001$; ***$p<0.0001$. Source data are provided as a Source Data file.

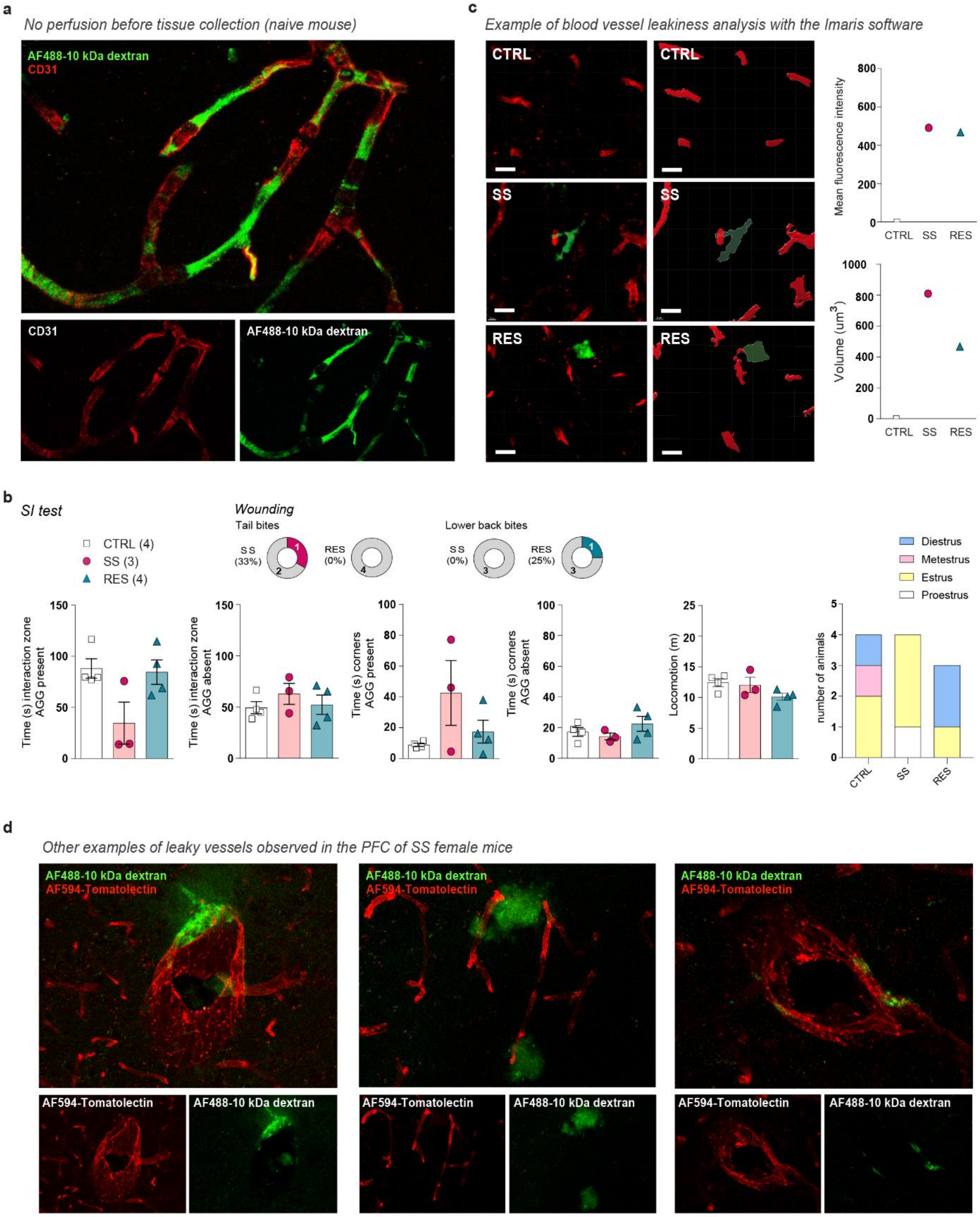

Supplementary Figure 8. Supplementary images and behavioural phenotyping for blood vessel leakiness in the prefrontal (PFC) of female mice. a, Dextran AlexaFluor488 (AF488-

dextran) could be detected in only PFC blood vessels of a naïve mouse 30 minutes after retro-orbital injection. No leakiness is observed in the brain parenchyma indicating intact BBB integrity. Images acquisition was performed on two mice. **b,** Additional graphs related to the social interaction test, wounding score and number of animals in each phase of the estrus cycle. **c,** Example of a quantitative BBB leakiness analysis performed with the Imaris software (right) for one animal per group on images acquired with a confocal microscope (left). **d,** Additional images of PFC BBB leakiness in female SS mice following CSDS, showing dye accumulation in the parenchyma and perivascular space of blood vessels. Image acquisition was performed on three mice. Data represent mean ± s.e.m; number of animals or subjects (*n*) is indicated on graphs. Multiple group comparisons were evaluated with two-way ANOVA followed by Bonferroni's multiple comparison test for estrus cycles (phase x phenotype) or one-way ANOVA followed by Bonferroni's multiple comparison test for other graphs. Source data are provided as a Source Data file

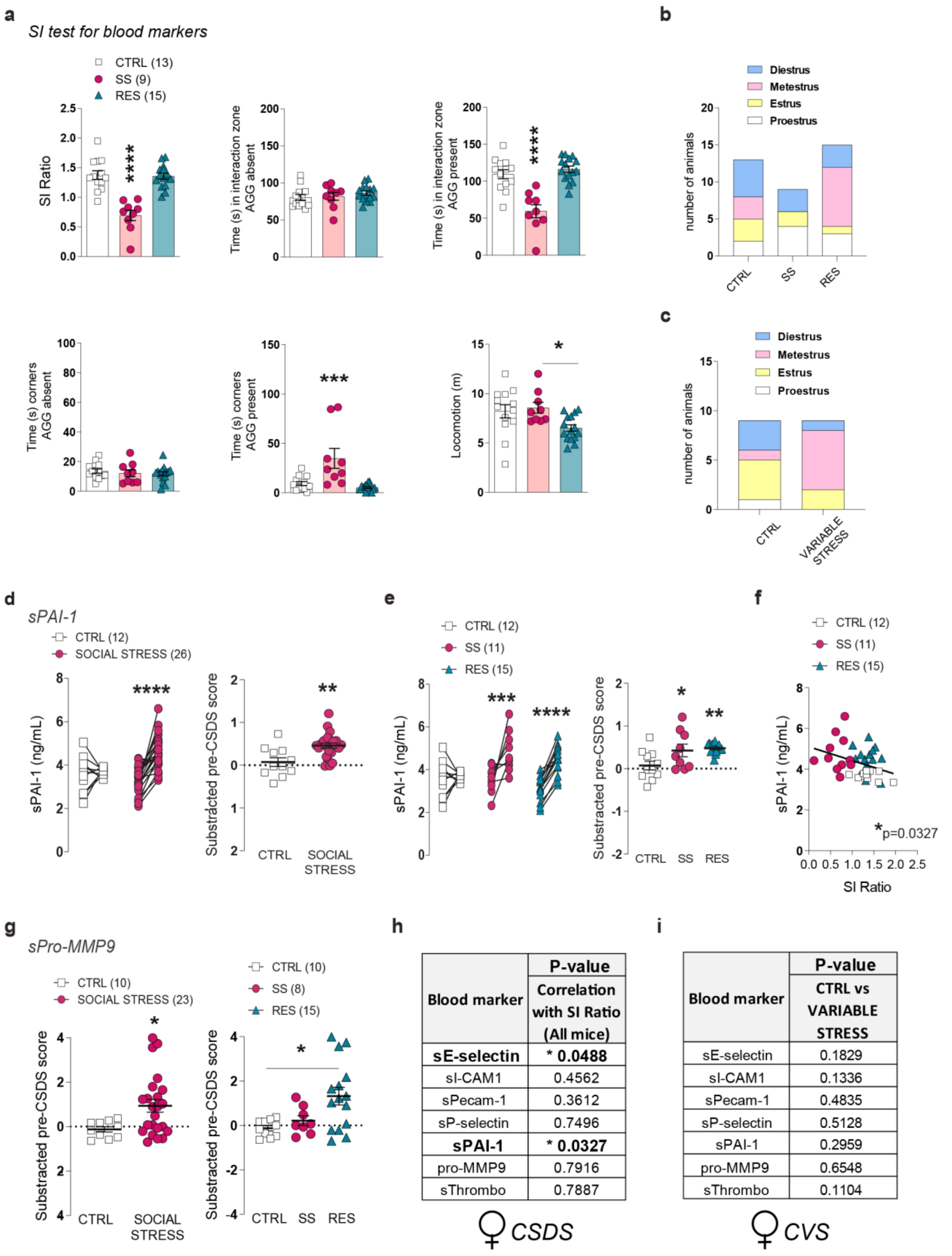

Blood marker	P-value Correlation with SI Ratio (All mice)
**sE-selectin**	*** 0.0488**
sI-CAM1	0.4562
sPecam-1	0.3612
sP-selectin	0.7496
**sPAI-1**	*** 0.0327**
pro-MMP9	0.7916
sThrombo	0.7887

Blood marker	P-value CTRL vs VARIABLE STRESS
sE-selectin	0.1829
sI-CAM1	0.1336
sPecam-1	0.4835
sP-selectin	0.5128
sPAI-1	0.2959
pro-MMP9	0.6548
sThrombo	0.1104

Supplementary Figure 9. Behavioural phenotype and supplemental data for Milliplex

experiments in female mice. a, Stress-susceptible (SS) mice spent less time in the interaction zone (****$p<0.0001$) and increased time in the corners (***$p=0.0003$) when the social target (aggressor, AGG) was present compared to unstressed control (CTRL) and resilient (RES) animals. No significant difference was observed when the social target was absent. RES animal travelled less distance (m) than SS mice in total (* $p=0.0163$). **b,** No significant difference was observed between phenotype and phase of the estrus cycle at the time of tissue collection for the chronic social defeat stress (CSDS) cohort (**b**) or the subchronic variable stress (SCVS) cohort (**c**). **d-f,** soluble plasminogen activator inhibitor 1 (sPAI-1) levels (ng/mL) were significantly increased in social stress (****$p<0.0001$, pre-CSDS score **$p=0.0016$), SS (**$p=0.003$, pre-CSDS score *$p=0.0478$) and RES (****$p<0.0001$, pre-CSDS score **$p=0.0069$) mice when compared to their baseline pre-CSDS levels and correlated with social avoidance (*$p=0.0327$). **g, s**Pro-MMP9 levels were significantly increased in social stress (*$p=0.0245$) and RES (**$p=0.0077$) animals following CSDS, when compared to their individual baseline levels. **h**, Individual correlation between serum levels of circulating vascular markers and SI Ratio was significant only for sE-selectin (*$p=0.0488$) and sPAI-1 (*$p=0.0327$) following CSDS. **i**, Circulating levels of vascular biomarkers following 6-d SCVS were not significantly different when compared to CTRL. Data represent mean ± s.e.m; number of animals or subjects (*n*) is indicated on graphs. 2-group comparisons were evaluated with unpaired t-tests, two-way ANOVA followed by Bonferroni's multiple comparison test for estrus cycles (phase x phenotype) and one-way ANOVA followed by Bonferroni's multiple comparison test for other graphs. Correlations were evaluated with Pearson's correlation coefficient and 2-group comparisons were evaluated with unpaired t-tests. *$p<0.05$; **$p<0.01$; ***$p<0.001$; ****$p<0.0001$. Source data are provided as a Source Data file.

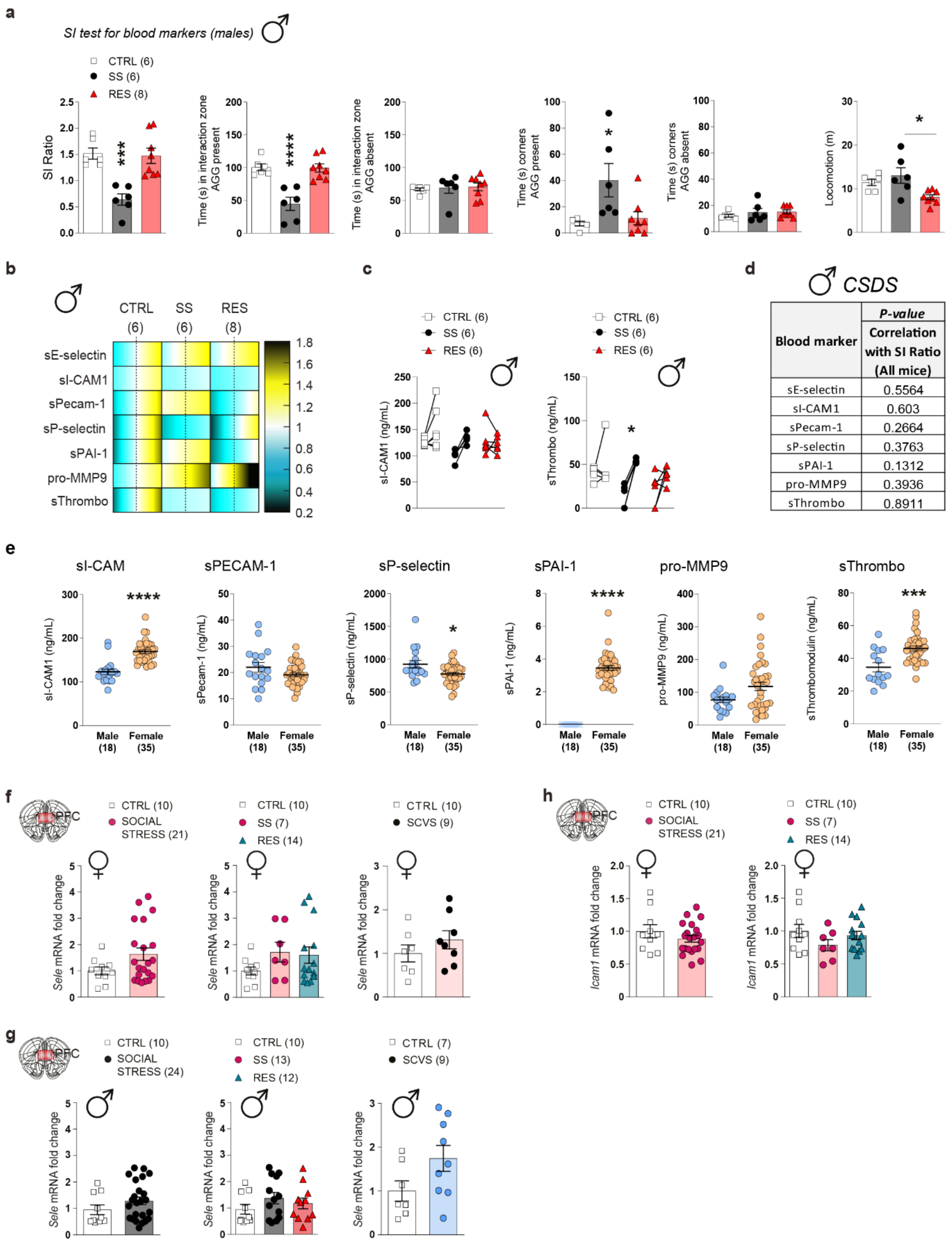

Supplementary Figure 10. Behavioural phenotype and supplemental data for Milliplex

experiments in male mice and baseline sex differences in circulating vascular markers. a, Stress-susceptible (SS) mice were characterized by lower social interaction (SI) ratio (***p=0.0003) with less time spent in the interaction zone (****p=0.0001) and increased time spent in the corners (*p=0.0146) when the social target (aggressor, AGG) was present compared to unstressed control (CTRL) and resilient (RES) animals. No significant difference was observed when the social target was absent. RES animal travelled less distance (m) than SS mice in total. **b,** Heatmap of circulating blood markers normalized to CTRL after 10-d chronic social defeat stress (CSDS). **c,** sThrombomodulin levels (ng/mL) significantly increased only in SS mice when compared to their individual baseline levels (*p=0.0304). **d,** None of the investigated markers correlated to social avoidance in male mice. **e,** Baseline sex-differences in circulating sI-CAM1 (****p<0.0001), sP-selectin (*p=0.0254), sPAI-1 (****p<0.0001) and sThrombomodulin (***p=0.0003) levels. *Sele* expression is increased without reaching significance in the PFC of stressed female (**f**) or male (**g**, SCVS: p=0.0795) mice. **h**, *Icam1* expression is reduced in the PFC of SS female without reaching statistical significance. Data represent mean ± s.e.m; number of animals or subjects (*n*) is indicated on graphs. 2-group comparisons were evaluated with unpaired t-tests and one-way ANOVA followed by Bonferroni's multiple comparison test for other graphs. *p<0.05; **p<0.01; ***p<0.001; ****p<0.0001. Source data are provided as a Source Data file

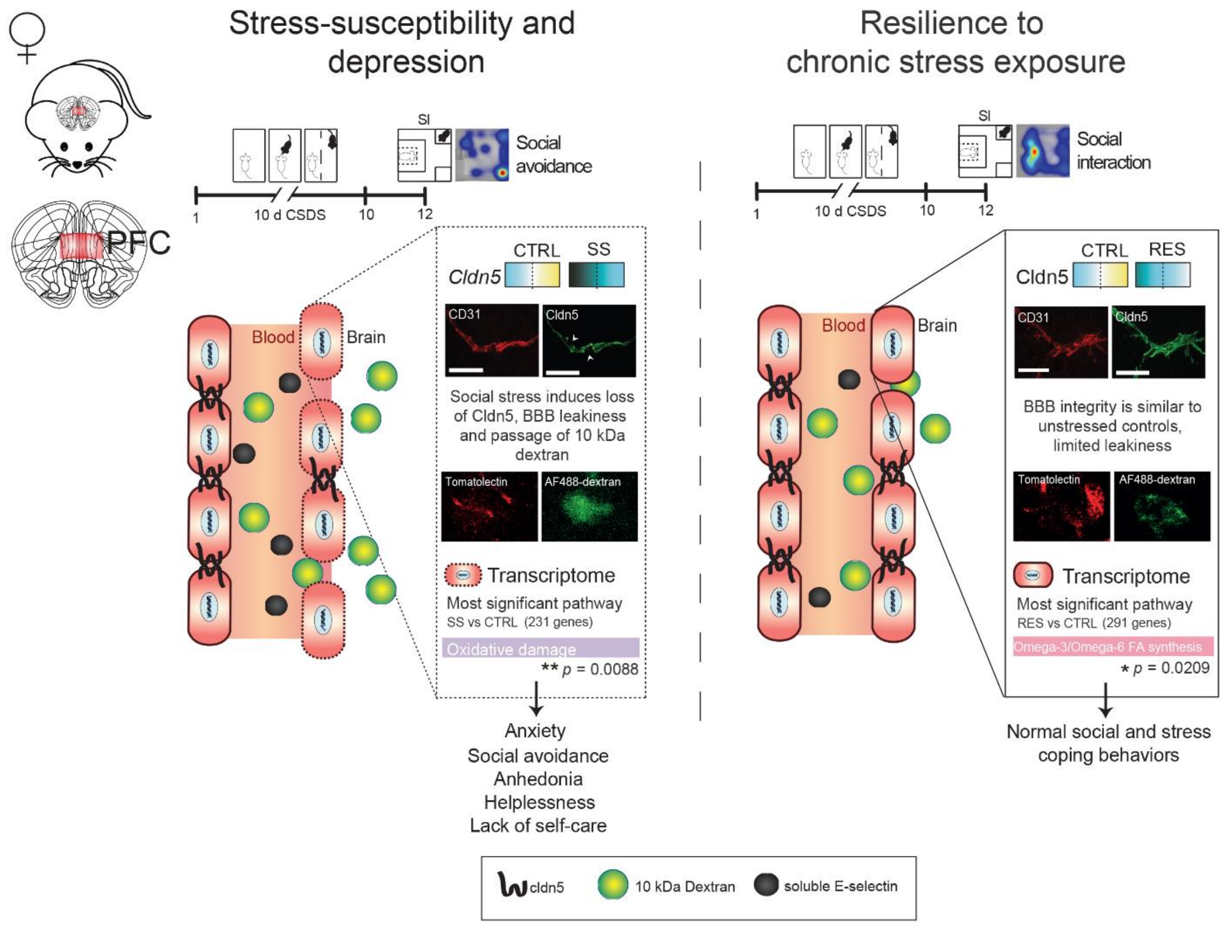

Supplementary Figure 11. Chronic stress induces blood-brain barrier (BBB) alterations in the female prefrontal cortex (PFC) promoting depressive behaviors vs resilience. 10-day chronic social defeat stress (CSDS) induces loss of Cldn5 expression at mRNA and protein levels in the PFC of stress-susceptible (SS) but not resilient (RES) female mice. Stress vulnerability is also associated with transcriptomic changes in the endothelium including for genes involved in oxidative damage. These molecular and cellular alterations are associated with anxiety and depression-like behaviors such as social avoidance, lack of self-care, anhedonia, and despair, as well as increased BBB leakiness favoring passage of circulating molecules from the bloodstream into the brain. Conversely, despite chronic stress exposure BBB integrity is maintained in the PFC of RES females, which display normal social and stress coping behaviors comparable to unstressed

controls (CTRL). This could be linked to transcriptomic adaptations and changes in the expression of endothelial genes related to omega/3-omega-6 fatty acid synthesis.

Supplementary Table 1. Mouse Primers qPCR

Gene	Ref Seq #	Assay ID	Forward primer	Reverse primer
Pecam1	NM_008816(2)	Mm.PT.58.43167370	PrimeTime®qPCR primers Exon Location 7-8	
Cdh5	NM_009868(1)	Mm.PT.58.8747496	PrimeTime®qPCR primers Exon Location 7-8	
Fgf2	NM_008006(1)	Mm.PT.56a.5129235	PrimeTime®qPCR primers Exon Location 1-3	
Angpt2	NM_007426(1)	Mm.PT.58.29139310	PrimeTime®qPCR primers Exon Location 1a-2	
Vegfa	NM_009505(3)	Mm.PT.58.14200306	PrimeTime®qPCR primers Exon Location 1-2	
Cldn1	NM_016674(1)	Mm.PT.58.41579102	PrimeTime®qPCR primers Exon Location 2-3	
Cldn3	NM_009902(1)	Mm.PT.58.43310459.g	PrimeTime®qPCR primers Exon Location 1-1	
Cldn5	NM_013805(1)	5’-TTTCTTCTATGCGCAGTTGG-3’	5’-GCAGTTTGGTGCCTACTTCA-3’	
Cldn12	NM_001193659(3)	Mm.PT.58.41535303	PrimeTime®qPCR primers Exon Location 3a-5	
Tjp1	NM_001163574(2)	Mm.PT.58.29459730	PrimeTime®qPCR primers Exon Location 18-19	
Tjp2	NM_001198985(2)	Mm.PT.58.16834535	PrimeTime®qPCR primers Exon Location 22-23	
Tjp3	NM_013769(1)	Mm.PT.58.43961106	PrimeTime®qPCR primers Exon Location 15-17	
Ocln	NM_008756(1)	Mm.PT.58.42749240	PrimeTime®qPCR primers Exon Location 7-9	
Marveld2	NM_001038602(2)	Mm.PT.58.7719303	PrimeTime®qPCR primers Exon Location 5-7	
Mfsd2a	NM_029662(1)	Mm.PT.58.32675283	PrimeTime®qPCR primers Exon Location 13-14	
Ntn1	NM_008744(1)	Mm.PT.58.41162316	PrimeTime®qPCR primers Exon Location 3-4	
Il1β	NM_008361(1)	Mm.PT.58.41616450	PrimeTime®qPCR primers Exon Location 3-4	
Il6	NM_031168(1)	Mm.PT.58.10005566	PrimeTime®qPCR primers Exon Location 4-5	
Tnfα	NM_013693(1)	Mm.PT.58.12575861	PrimeTime®qPCR primers Exon Location 2-4	
Cxcl1	NM_008176(1)	Mm.PT.58.42076891	PrimeTime®qPCR primers Exon Location 2-4	
Cxcl12	NM_013655(3)	Mm.PT.58.8366748	PrimeTime®qPCR primers Exon Location 1-2	
Ccl2	NM_011333(1)	Mm.PT.58.42151692	PrimeTime®qPCR primers Exon Location 1-3	
Ccl3	NM_011337(1)	Mm.PT.58.29283216	PrimeTime®qPCR primers Exon Location 1-2	
Ccr2	NM_009915(1)	Mm.PT.58.14116710	PrimeTime®qPCR primers Exon Location 1-3	
Gapdh	NM_008084(1)	Mm.PT.39a.1	PrimeTime®qPCR primers Exon Location 2-3	
Nostrin	NM_181547(1)	Mm.PT.58.12259437	PrimeTime®qPCR primers Exon Location 14-16	
Aldh1h1	NM_027406(1)	Mm.PT.58.7775479	PrimeTime®qPCR primers Exon Location 13-15	
Aqp4	NM_009700(1)	Mm.PT.58.9080805	PrimeTime®qPCR primers Exon Location 1-2	
Mbp	NM_001025254 (4)	Mm.PT.58.28532164	PrimeTime®qPCR primers Exon Location 6-9	
Slc17a6	Mbp NM_080853(1)	Mm.PT.58.10363705	PrimeTime®qPCR primers Exon Location 9-10	
Sele	NM_011345(1)	Mm.PT.58.29497526	PrimeTime®qPCR primers Exon Location 13-14	

Supplementary Table 2. Human Primers qPCR

Gene	Ref Seq #	Assay ID	Primers
CLDN5	NM_003277(2)	Hs.PT.58.1483777.g	PrimeTime®qPCR primers Exon Location 1-1
GAPDH	NM_002046(1)	Hs.PT.39a.22214836	PrimeTime®qPCR primers Exon Location 2-3

Supplementary Table 3. Complete demographic for human brain cohort

Age of the individuals (years): 25-85

Postmortem interval (hours): 2.5-132.68

Gender	Cause of death<br>(Depressive symptoms at the time)	Antidepressant treatment
F	Accident (no)	No
F	Natural (no)	No
F	Suicide (no)	Yes
F	Natural (no)	No
F	Accident (no)	No
F	Accident (no)	Yes
F	Natural (no)	No
F	Accident (no)	No
F	Accident (no)	No
F	Accident (no)	Yes
F	Natural (no)	Yes
F	Undetermined (no)	No
F	Accidental (no)	No
F	Natural (no)	No
F	Natural (no)	No
F	Suicide (yes)	No
F	Suicide (yes)	No
F	Suicide (yes)	No
F	Suicide (yes)	No
F	Suicide (yes)	No
F	Suicide (yes)	No
F	Natural (yes)	No
F	Suicide (yes)	No
F	Suicide (yes)	No
F	Suicide (yes)	No
F	Suicide (yes)	No
F	Suicide (yes)	No
F	Suicide (yes)	Yes
F	Suicide (yes)	Yes
F	Suicide (yes)	Yes
F	Suicide (yes)	Yes
F	Suicide (yes)	Yes
F	Suicide (yes)	Yes
F	Suicide (yes)	Yes
F	Suicide (yes)	Yes

F	Accidental (yes)	Yes
F	Suicide (yes)	Yes
F	Natural (yes)	Yes
F	Suicide (yes)	Yes
F	Suicide (yes)	Yes
F	Natural (yes)	Yes
F	Natural (yes)	Yes
F	Suicide (yes)	Yes

Supplementary Table 4. Complete demographic data for human serum cohort

Age of the participants (years): 18-30

Gender	Depressive symptoms	Current suicidal thoughts
F	No	No
F	No	No
F	No	No
F	No	No
F	No	No
F	No	No
F	No	No
F	No	No
F	No	No
F	No	No
F	No	No
F	No	No
F	No	No
F	No	No
F	No	No
F	No	Yes
F	No	No
F	No	No
F	No	No
F	No	No
F	No	No
F	No	No
F	Yes	No
F	Yes	No
F	Yes	No
F	Yes	Yes
F	Yes	Yes
F	Yes	Yes
F	Yes	Yes
F	Yes	Yes
F	Yes	No
F	Yes	N/A
F	Yes	N/A
F	Yes	N/A
F	Yes	N/A
F	Yes	N/A

F	Yes	N/A
F	Yes	N/A
F	Yes	N/A
F	Yes	N/A
F	Yes	No
F	Yes	Yes
F	Yes	Yes
F	Yes	Yes
F	Yes	No
F	Yes	No
F	Yes	No
F	Yes	No
F	Yes	No
F	Yes	Yes
M	No	No
M	No	No
M	No	No
M	No	No
M	No	No
M	No	No
M	No	No
M	No	No
M	No	No
M	No	No
M	No	No
M	No	No
M	Yes	No
M	Yes	Yes
M	Yes	Yes
M	Yes	Yes
M	Yes	No
M	Yes	Yes
M	Yes	No
M	Yes	Yes
M	Yes	No
M	Yes	Yes
M	Yes	Yes
M	Yes	No
