# Supplementary material for: Vascular and blood-brain barrier-related changes underlie stress responses and resilience in female mice and depression in human tissue
Source: Nat Commun. 2022 Jan 10;13:164. doi: 10.1038/s41467-021-27604-x (PMC8748803; doi:10.1038/s41467-021-27604-x)
Supplement: Supplementary file 3 — Reporting Summary [file 41467_2021_27604_MOESM3_ESM.pdf]

nature research

Corresponding author(s): Caroline Ménard, PhD

Last updated by author(s): Oct 29, 2021

Reporting Summary

Nature Research wishes to improve the reproducibility of the work that we publish. This form provides structure for consistency and transparency in reporting. For further information on Nature Research policies, see our Editorial Policies and the Editorial Policy Checklist.

Statistics

For all statistical analyses, confirm that the following items are present in the figure legend, table legend, main text, or Methods section.

n/a	Confirmed	
☐	☒	The exact sample size (n) for each experimental group/condition, given as a discrete number and unit of measurement
☐	☒	A statement on whether measurements were taken from distinct samples or whether the same sample was measured repeatedly
☐	☒	The statistical test(s) used AND whether they are one- or two-sided<br>*Only common tests should be described solely by name; describe more complex techniques in the Methods section.*
☒	☐	A description of all covariates tested
☐	☒	A description of any assumptions or corrections, such as tests of normality and adjustment for multiple comparisons
☐	☒	A full description of the statistical parameters including central tendency (e.g. means) or other basic estimates (e.g. regression coefficient) AND variation (e.g. standard deviation) or associated estimates of uncertainty (e.g. confidence intervals)
☐	☒	For null hypothesis testing, the test statistic (e.g. F, t, r) with confidence intervals, effect sizes, degrees of freedom and P value noted<br>*Give P values as exact values whenever suitable.*
☒	☐	For Bayesian analysis, information on the choice of priors and Markov chain Monte Carlo settings
☒	☐	For hierarchical and complex designs, identification of the appropriate level for tests and full reporting of outcomes
☐	☒	Estimates of effect sizes (e.g. Cohen's d, Pearson's r), indicating how they were calculated

Our web collection on statistics for biologists contains articles on many of the points above.

Software and code

Policy information about availability of computer code

Data collection	When possible, behavioral tests were recorded and analyzed using AnyMaze software (AnyMaze™ 6.1 Stoelting Co.).
Data analysis	Normality was determined by D'Agostino–Pearson, Shapiro–Wilk and Kolmogorov–Smirnov normality tests using GraphPad Prism software (version 9.0). Most datasets were normally distributed, and then t-tests, one-way ANOVAs, two-way ANOVAs, and Pearson's correlations were performed with GraphPad Prism software (version 9.0). Bonferroni was used as a post hoc test when appropriate for one-way and two-way ANOVAs and statistical significance was set at p<0.05. If datasets were not normally distributed a non-parametric Mann-Whitney or Kruskal-Wallis test was used for two or three groups, respectively. Statistical significance was set at p<0.05 with *p<0.05; **p<0.01; ***p<0.001; ****p<0.0001. Values between p=0.05 and p<0.10 were considered as trending without reaching significance.<br>FACS analyses were performed on BD LSR II and data were analyzed with FACS Diva software (BD Biosciences, v.6.1.3) and FlowJo software (Tree Star, v.10.5.3). Confocal images were analyzed using Imaris 9.6.1 software. Gene expression profiling was performed on Thermofisher Transcriptome Analysis Console 4.0.

For manuscripts utilizing custom algorithms or software that are central to the research but not yet described in published literature, software must be made available to editors and reviewers. We strongly encourage code deposition in a community repository (e.g. GitHub). See the Nature Research guidelines for submitting code & software for further information.

Data

Policy information about availability of data

All manuscripts must include a data availability statement. This statement should provide the following information, where applicable:

- Accession codes, unique identifiers, or web links for publicly available datasets
- A list of figures that have associated raw data
- A description of any restrictions on data availability

All data supporting the findings of this study are available within the paper and Supplementary Information files. RNA sequencing datasets for male and female mouse PFC endothelium have been deposited in the GEO publicly accessible database under the accession code SuperSeries GSE173823. Raw sequencing data for the male mouse NAc endothelium are available in the Supporting Information of Dudek et al. (2020). RNA sequencing datasets for human tissue sets have been deposited in the GEO under the accession code GSE102556 as reported in Labonte et al. (2017). Source data are provided with this paper.

Field-specific reporting

Please select the one below that is the best fit for your research. If you are not sure, read the appropriate sections before making your selection.

[x] Life sciences [ ] Behavioural & social sciences [ ] Ecological, evolutionary & environmental sciences

For a reference copy of the document with all sections, see nature.com/documents/nr-reporting-summary-flat.pdf

Life sciences study design

All studies must disclose on these points even when the disclosure is negative.

Sample size	Sample size for chronic social defeat stress (CSDS) and subchronic variable stress (SCVS) mouse cohorts were calculated based on previous studies of CSDS, SCVS and depression-like behaviors (Golden et al., 2013; Hodes et al., 2014; Hodes et al., 2015; Menard et al., 2017). Sample size for viral experiments was determined based on a previous study performed in male mice (Menard et al., 2017). For transcriptomic experiments, no statistical method was used to determine the required sample size, it was based on similar experiments conducted in male mice (Menard et al., 2017; Dudek et al., 2020). For human tissue experiments (postmortem brain tissue and blood biomarkers), no statistical method was used to determine the required sample size. Human brain and serum samples were provided by the Douglas-Bell Brain Bank and the Signature Biobank, respectively, and we have used all samples available at the time to avoid bias.
Data exclusions	Exlusion criteria was pre-established before conducting experiments. Outliers for social interaction test screening were identified as being greater than two standard deviations from the mean and excluded. In chronic social defeat stress (CSDS), all mice were assigned to stress-susceptible (SS) or resilient (RES) groups based on their behavioral profile when compared to unstressed controls (CTRL). Outliers for behavioral testing, for example characterized by impaired locomotion, were identified as being greater than two standard deviations from the mean and excluded from statistical analysis. An animal found to be an outlier in more than 2 behavioral tests was removed from all analysis.
Replication	All paradigms (CSDS, SCVS, viral injections) were performed in at least two cohorts of mice to ensure reproductibility of results (i.e two cohorts of CSDS were performed for quantitative PCR experiments, etc.). Replication was successful in every case. At least 3 animals or subjects per experimental groups were used (ex: for immunohistochemistry quantification). When automatic tracking was not available, blind observers analyzed behavioral data to avoid bias.
Randomization	All mice were assigned to stress-susceptible (SS) or resilient (RES) groups based on their behavioral profile when compared to unstressed controls (CTRL). For chronic social defeat stress (CSDS), subchronic variable stress (SCVS) and viral injections, mice were randomly assigned to CTRL or experimental group upon arrival. For human tissue experiments, samples were provided by the Douglas-Bell Brain Bank (brain tissue) and the Signature Biobank (blood) and subjects were already allocated to CTRL or MDD groups based on clinical records and interviews obtained and reviewed by mental health professionals to establish independent diagnoses in line with the Diagnostic and Statistical Manual of Mental Disorders (DSM) IV criteria. Covariates were not controlled.
Blinding	Social interaction test screening and behavioral tests were performed with automated tracking systems when possible. If not, scoring was done by experimenters blinded to experimental conditions (for splash test, sucrose preference test and forced swim test). For immunohistochemistry quantification, sample IDs were renamed to render the experimenter 'blind', and true subject IDs were revealed once data analysis was finished.

Reporting for specific materials, systems and methods

We require information from authors about some types of materials, experimental systems and methods used in many studies. Here, indicate whether each material, system or method listed is relevant to your study. If you are not sure if a list item applies to your research, read the appropriate section before selecting a response.

Materials & experimental systems

n/a	Involved in the study	
☐	☒	Antibodies
☒	☐	Eukaryotic cell lines
☒	☐	Palaeontology and archaeology
☐	☒	Animals and other organisms
☐	☒	Human research participants
☒	☐	Clinical data
☒	☐	Dual use research of concern

Methods

n/a	Involved in the study	
☒	☐	ChIP-seq
☐	☒	Flow cytometry
☒	☐	MRI-based neuroimaging

Antibodies

Antibodies used

Primary antibodies: Rabbit anti-cldn5, 1:250, Life Technologies, #34-1600; Rat anti-CD31, 1:150, Invitrogen, #14-0311-85; Secondary antibodies: anti-rabbit-Cy2, 1:400, Jackson Immunoresearch, #711-225-152; anti-rat-Cy3, 1:400, Jackson Immunoresearch, #712-165-153; Lycopersicon Esculentum (Tomato) Lectin DyLight® 594, 1:400, Vector Labs, L32471

Validation

Primary antibodies were used according to the following conditions and references : Rabbit anti-cldn5 and Rat anti-CD31 (Menard et al., 2017; Campbell et al., 2008; Doyle et al., 2014; Keaney et al., 2015); Lycopersicon Esculentum (Tomato) Lectin DyLight® 594 (Lehmann et al., 2018)

Animals and other organisms

Policy information about studies involving animals; ARRIVE guidelines recommended for reporting animal research

Laboratory animals

Female and male C57BL/6J mice (about 20 g) were purchased at 7 weeks of age from Charles River and allowed one week of acclimation to the housing facility of CERVO Brain Research Center before starting the experiments. Sexually experienced retired male and female CD-1 breeders (about 40 g) of at least 4 months of age were purchased from Charles River Laboratories and used as aggressors or social interaction (SI) targets. All mice were single housed following CSDS or group housed during SCVS and maintained on a 12- h/12-h light/dark cycle throughout. Room temperature was maintained between 19 and 23°C and humidity kept around 40-45%. Mice were provided with ad libitum access to water and food. All mouse procedures were performed in accordance with the Canadian Council on Animal Care as well as the Animal Care and use of Université Laval.

Wild animals

No wild animals were used.

Field-collected samples

No samples collected from the field

Ethics oversight

All mouse procedures were performed in accordance with the Canadian Council on Animal Care, Animal care and use committee of Université Laval (Certificate #2019-VRR-18-052), National Institutes of Health Guide for Care and Use of Laboratory Animals as well as the Icahn School of Medicine at Mount Sinai Animal Care and Use Committee.

Note that full information on the approval of the study protocol must also be provided in the manuscript.

Human research participants

Policy information about studies involving human research participants

Population characteristics

Whole-tissue NAc or PFC resections were collected and provided by the Quebec Suicide Brain Bank at the Douglas Hospital Research Center (McGill University) under approval of the institution's Ethics Committee. Brain tissue was collected at local medical examiners' office after obtaining permissions. Blood toxicology was performed to exclude subjects using illicit drugs or psychotropic medications. Subjects with a known history of neurological disorders or head injury were also excluded. Demographic characteristics associated with each sample are listed in Supplementary Table 3. Clinical records and interviews were obtained for each case and reviewed by three or four mental health professionals to establish independent diagnoses followed by a consensus diagnosis in line with the Diagnostic and Statistical Manual of Mental Disorders (DSM) IV criteria. Cohorts were matched as closely as possible for gender, age, race, pH, post-mortem interval, and RNA integrity number (Menard et al., 2017; Golden et al., 2013).
Human blood samples from healthy and depressed volunteers were collected and provided by Signature Bank from the Centre de recherche de l'Institut universitaire en santé mentale de Montréal (CR-IUSMM) under approval of the institution's Ethics Committee. Subjects with known history of drug abuse were excluded. Demographic characteristics associated with each sample are listed in Supplementary Table 4. Depressive behaviors were assessed by the Patient Health Questionnaire (PHQ-9), which scores each of the nine Diagnostic and Statistical Manual of Mental Disorders (DSM) IV criteria

All experiments were performed under the approval of Université Laval and CERVO Brain Research Center Ethics Committee Neurosciences et santé mentale (Project #2019-1540).

Recruitment

N/A (all samples were provided by banks)

Ethics oversight

Post-mortem brain tissue was provided by the Quebec Suicide Brain Bank at the Douglas Hospital Research Center (McGill University) under approval of the institution's Ethics Committee. Human blood samples were collected and provided by the

Signature Bank from the Centre de recherche de l'Institut universitaire en santé mentale de Montréal (CR-IUSMM) under approval of the institution's Ethics Committee. All donors provided informed consent and signed a 7-page document detailing the goals of the Signature Bank, participants involvement (questionnaires and tissue sampling), advantages vs risks, compensation, confidentiality measures, rights as participant and contact information. All experiments were performed under the approval of Université Laval and CERVO Brain Research Center Ethics Committee Neurosciences et santé mentale (Project #2019-1540).

Note that full information on the approval of the study protocol must also be provided in the manuscript.

Flow Cytometry

Plots

Confirm that:

- [x] The axis labels state the marker and fluorochrome used (e.g. CD4-FITC).
- [x] The axis scales are clearly visible. Include numbers along axes only for bottom left plot of group (a 'group' is an analysis of identical markers).
- [x] All plots are contour plots with outliers or pseudocolor plots.
- [x] A numerical value for number of cells or percentage (with statistics) is provided.

Methodology

Sample preparation	Bilateral 2mm punches from region of interest were collected from two adjacent 1 mm coronal slices on wet ice after rapid decapitation and immediately processed for MACS purification (Dudek et al., 2019). Endothelial cells were enriched from punches by using MACS according to the manufacturer's protocol (Miltenyi Biotec). Briefly, brain punches were dissociated using a neuronal tissue dissociation kit (Miltenyi Biotec, 130-092- 628), applied on a 70-µm MACS smart strainer and washed with HBSS 1×. Thereafter, cells were magnetically labeled with CD45 microbeads (Miltenyi Biotec, 130-052-301) and passed through a MACS MS column (Miltenyi Biotec, 130-042-201) to proceed to negative selection of CD45 cells. CD45− fraction was collected and magnetically labeled with CD31 microbeads (Miltenyi Biotec, 139097418) and then passed through MACS MS column to positively select CD31+ cells.<br>Then, original, CD45−, CD45+, CD45− CD31−, and CD45− CD31+ cell fraction aliquots were incubated with anti-CD16/32 (BioLegend, 14-0161-82) to block Fc receptors. Cells were then labeled with CD45 APC (BioLegend, 103111) and CD31 PE-CF594 (BD Biosciences, 653616). A viability dye (LIVE/DEAD fixable green, Molecular Probes, L34969) was added to the previous panels to discriminate live cells. Endothelial cells from mouse brain punches were identified as CD45− and CD31+ cells.
Instrument	BD FACS AriaII SORP
Software	Data were collected using BD FACSDiva software and analyzed with FlowJo software (Tree Star).
Cell population abundance	Post-sort fractions were not used in this study
Gating strategy	Endothelial cells were gated as single live CD31+/CD45- as reported in Dudek et al., PNAS (2020).

- [x] Tick this box to confirm that a figure exemplifying the gating strategy is provided in the Supplementary Information.
